# Magnetite Impregnated Lignocellulosic Biomass for Zn(II) Removal

**DOI:** 10.3390/ma15030728

**Published:** 2022-01-19

**Authors:** Christopher Asimbaya, Nelly Maria Rosas-Laverde, Salome Galeas, Alexis Debut, Victor H. Guerrero, Alina Pruna

**Affiliations:** 1 Department of Materials, Escuela Politécnica Nacional, Quito 170524, Ecuador; christopher.gab26@gmail.com (C.A.); nelly.rosas@epn.edu.ec (N.M.R.-L.); salome.galeas@epn.edu.ec (S.G.); 2 Centro de Nanociencia y Nanotecnología, Universidad de las Fuerzas Armadas (ESPE), Sangolqui 171103, Ecuador; apdebut@espe.edu.ec; 3 Institute of Materials Technology, Universitat Politècnica de València, 46022 Valencia, Spain; 4 Center for Surface Science and Nanotechnology, University Politehnica of Bucharest, 313 Splaiul Independentei, 060042 Bucharest, Romania

**Keywords:** aqueous synthetic solutions, magnetite nanoparticles, adsorption isotherms, diffusion kinetic models, physisorption

## Abstract

Magnetic composites obtained by impregnation of lignocellulosic biomass with magnetite nanoparticles were used for zinc(II) removal from aqueous synthetic solutions. Laurel, canelo and eucalyptus sawdust, with a particle size between 74 and 150 µm were used as support. Structural and morphological examinations of the composites confirmed the presence of magnetite nanoparticles in the lignocellulosic support. Transmission Electron Microscopy showed nanoparticles with diameters of about 20 nm. The maximum removal efficiencies for 7 g L^−1^ of modified adsorbent were increased to 98.9, 98.8 and 97.6% for laurel, canelo and eucalyptus magnetic composites, respectively, in comparison to 60.9, 46.0 and 33.3%, for corresponding unmodified adsorbents. Adsorption data was analyzed using pseudo-first, pseudo-second order and intra-particle diffusion kinetic models and various isotherm models. The results determined that Freundlich isotherm fits the Zn ions adsorption on magnetite modified adsorbents while chemisorption and boundary diffusion were dominating the process.

## 1. Introduction

The increasing industrial activity during the last decades has resulted in a worldwide concern problem: heavy metals contamination of liquid effluents. These metals have become a serious problem because their ions are not biodegradable and accumulate in animal tissues, damaging human health and water quality [1,2,3]. One of the most common heavy metals in industrial discharges is zinc, and the main sources of pollution are the automotive, electroplating, iron and steel industries [2,4,5,6].

Nowadays, several techniques are used to remove heavy metals from water, such as: sedimentation, filtration, electrocoagulation, ion exchange and reverse osmosis, however, disadvantages of these methods are low efficiency, high energy requirements, incompatibility with pollutants and toxic residues generation, that require subsequent treatments [6,7]. For these reasons, alternative treatments have been developed and optimized in the few last decades. One of the most efficient, low cost, that is widely used is adsorption [8,9].

The most common adsorbent used for heavy metal removal is activated carbon, however it is expensive. Recent studies focus on alternative materials: inexpensive, abundant in nature, with minimal processing requirement and in some cases by-products of other industries [9]. These materials include waste wood, mainly sawdust, which due to its porosity has high free surface that results in removal of heavy metals through the formation of bonds between the metal ions and the oxygen of ionizable functional groups [10].

Therefore, nanotechnology has allowed the development of more efficient adsorbent materials for environmental remediation. For example, iron nano-oxides (magnetite, hematite and maghemite nanoparticles) have a magnetic core that improve the adsorption process. In addition, they allow an easy recovery, handling and less contamination [11]. This, combined with the adsorptive capacity of the sawdust allows the removal of metal ions from liquid effluents to be more effective and the recovery of the residue to be easier and less expensive [12].

Numerous studies performed during the last decades have reported lignocellulosic materials impregnated with magnetic materials that have been used successfully to remove metals from contaminated water. Moafi et al. [13] used a wood sawdust/hematite (α-Fe_2_O_3_) nanocomposite to adsorb arsenic (III) from water with a removal capacity of 58.8 mg g^−1^. Panneerselvam et al. [5] obtained a nickel removal of 38.3 mg g^−1^ using tea residues impregnated with magnetic nanoparticles. Additionally, Gupta and Nayak [14] used orange peel residues impregnated with magnetite nanoparticles to treat cadmium-contaminated effluents and reported an adsorption capacity of 76.9 mg g^−1^.

Wood residues are widely available around the globe, are cheap and have a particularly favorable composition, structural and morphological characteristics for adsorption. For instance, Ecuador has become one of the most biodiverse countries around the world due to the variety of climates which resulted in high availability of plant species. According to National Financial Corporation [15] statistics for 2016, forest area of Ecuador was around 54,000 ha, and the annual production of wood was about 421,000 metric tons. Additionally, wood industry is well known for generating big amounts of waste, up to 70% of the wood raw materials can become chippings, sawdust or off-cuts [16,17]. Therefore, wood residues can be advantageously used to remove a wide variety of contaminants from aqueous solutions [18]. Although the removal of heavy metals using magnetic composite bioadsorbents has been investigated by several previous authors, the use of supporting materials derived from species such as Ecuadorian laurel (Cordia alliodora), canelo (Ocotea javitensis) and eucalyptus (Eucalyptus glodulus) has not been sufficiently analyzed. In several studies, the improvement of the adsorptive performance obtained by using particulated materials to impart magnetic properties to the adsorbents is not well defined and understood. Moreover, in some cases the study of the kinetics and equilibrium processes associated to the adsorptive removal of pollutants using this type of materials is limited to fit the experimental data obtained to a particular set of models without verifying the validity and applicability of the underlying assumptions [19]. For this reason, in this work, we studied the absorptive removal of zinc(II) ions from synthetic wastewaters by using the previously mentioned wood residues to obtain magnetic adsorbents by magnetite nanoparticles impregnation. The obtained adsorbents were characterized by using several analytical techniques and the influence of the magnetite nanoparticles on the removal performance of the composites was determined. The experimental data obtained in batch tests was fitted to a series of isotherm and kinetics models, aiming to elucidate the nature and the factors that affect the ability of the composites for capturing the metal ions in the solution.

## 2. Materials and Methods

### 2.1. Materials

Iron(III) chloride hexahydrate (FeCl_3_∙6H_2_O) (LOBA Chemie, Mumbai, India), iron(II) sulfate heptahydrate (FeSO_4_∙7H_2_O) (Panreac, Barcelona, Spain) and ammonium hydroxide solution (NH_4_OH, 29%) (Fisher Chemicals, New York, NY, USA). These reagents were used to obtain magnetic nanoparticles. Zinc chloride (ZnCl_2_) (Merck, Kenilworth, NJ, USA) was used to prepare Zn^2+^ synthetic solutions for removal tests. All reagents were of analytical grade. The solutions were prepared with pure water (Type 2). Lignocellulosic residues (laurel, yellow canelo and eucalyptus wood chips) were obtained from a local sawmill in Quito, Ecuador.

### 2.2. Lignocellulosic Biomass Conditioning and Characterization

Wood chips of laurel, canelo and eucalyptus (separately) were washed several times with water and dried at 100 °C for 12 h, then reduced up to 0.5 mm with a knife-mill (Thomas Scientific, model 3379-K05, Philadelphia, PA, USA), and classified by sieving (vibratory sieve shaker, Retsch, AS 200 basic, Llanera, Spain) for 30 min in order to retain the fraction between 74 and 150 µm to be used as support of the magnetic nanocomposites.

Chemical composition of lignocellulosic residues was determined using ASTM standards methods: D1106-96 (lignin), D1109-84 (hemicellulose), ASTM D1110-84 and D1107-96 (extractive components). Cellulose content was determined by difference. Moisture, ash and volatile matter content were measured by ASTM standards (D4442-07, D1102-84 and ASTM E872–82, respectively, ASTM International, Lima, Peru). Fixed carbon content was determined by difference. Functional groups of lignocellulosic residues were identified by Fourier Transform Infrared Spectroscopy (FTIR, Perkin Elmer, São Paulo, Brazil, Spectrum 100/Spotlight 200, range: 4000 to 500 cm^−1^). Surface morphology and particle size were determined by Scanning Electron Microscopy (SEM, Tescan, Mira 3, Quito, Ecuador) and Transmission Electron Microscopy (TEM, FEI, G2 Spirit Twin, Madrid, Spain), respectively.

### 2.3. Magnetite Impregnation of Lignocellulosic Biomass and Characterization

Magnetite nanoparticles were synthesized by controlled precipitation method. FeCl_3_∙6H_2_O (6.1 g) and FeSO_4_∙7H_2_O (4.2 g) were added to 100 mL of water, heated to 35 °C in an argon atmosphere with stirring (1250 rpm). Next, 10 mL of 29% ammonia solution was added and the reaction was carried out for 30 min. Finally, the lignocellulosic residue was added to the resultant solution keeping it at 35 °C and constant stirring for another 30 min (magnetite/residue ratio: 1:1). The obtained black precipitate was naturally cooled down to room temperature and thoroughly rinsed with pure water until the unreacted reagents were removed and a neutral pH of the solution was reached. The precipitate was magnetically separated from the solution and dried at 70 °C for 12 h. This procedure was performed for each type of wood.

Magnetite impregnated sorbents were characterized by X-ray Diffraction (Panalytical, Empyrean XRD with a Cu Kα source, 40 kV, 40 mA, 2θ range: 10 to 90°, Quito, Ecuador), FTIR microscopy in ATR mode (Perkin Elmer, Spectrum 100 Spectrometer with Spotlight 200 microscope, São Paulo, Brazil), SEM (Tescan, Mira 3, Quito, Ecuador) and TEM (FEI, G2 Spirit Twin, Madrid, Spain) microscopy.

### 2.4. Adsorption Studies

Batch removal tests were performed at 298 K in order to optimize experimental conditions. Each of the obtained nanocomposites were added to zinc chloride synthetic solutions (ZnCl_2_) at constant stirring (150 rpm, orbital shaker KS 130, IKA, Staufen, Germany) and pH was adjusted at 6 [20], by addition of 0.1 M NaOH or HCl solutions. Zinc concentration after adsorption was analyzed by visible spectroscopy (HACH, DR1900, Loveland, CO, USA) as established in the APHA 3500—Zn method. All the tests were performed in duplicate [21].

The optimal contact time was determined using 50 mg L^−1^ zinc precursor solutions with adsorbent dosage of 5 g L^−1^. Samples were collected at 30, 45, 60, 120, 180, 240 and 300 min in order to determine evolution of removal process. Then, using the optimal contact time (3 h), the effect of adsorbent dosage on zinc removal was studied. The magnetite modified adsorbents were added to 50 mg L^−1^ zinc solutions at a dosage of 3, 5, 7 and 10 g L^−1^, at pH value of 6. Non-modified adsorbents were employed as reference samples, in the same conditions.

Adsorption isotherms were obtained using the optimal adsorbent dosage and contact time. Zinc chloride solutions of 25, 50, 100, 150, 200 and 300 mg L^−1^ [22] were used. The experiments were carried out at 298 K, constant stirring at 150 rpm and pH 6.

For the adsorption experiments, the following equation was employed to calculate the equilibrium adsorption capacity, $q_{e}$ (mg g^−1^) of adsorbent:

(1) $$q_{e}=V\left(C_{o}-C_{e}\right)/m,$$

where V (L) is the solution volume, $C_{o}$ and $C_{e}$ (mg L^−1^) are the initial and equilibrium concentrations of Zn solutions, respectively, and m (g) is the mass of dry sorbent. The removal efficiency is calculated as a ratio between adsorbed value at time t and the initial zinc concentration (mg L^−1^).

The Equations (2)–(4) for pseudo first-order (PFO) model (Equation (2)), pseudo second-order (PSO) model (Equation (3)) and intra-particle diffusion model [23] (Equation (3)):

(2) $$q\left(t\right)=q_{e}\left(1-e^{-k_{1}t}\right),$$

(3) $$t/q\left(t\right)=1/\left(k_{2}q_{e}^{2}\right)+t/q_{e}$$

(4) $$q\left(t\right)=k_{p}t^{0.5}+C$$

were used to analyze the kinetic data, where q(t) (mg g^−1^) is the amount of Zn adsorbed on the sorbent at any time t (h), k_1_ (min^−1^), k_2_ (g (mg min)^−1^) and k_p_ (g mg^−1^ min^−0.5^) are the PFO, PSO and intraparticle diffusion kinetic model constants, respectively.

The adsorption isotherm data was fitted with Langmuir, Freundlich and Temkin models in their linearized forms as described by Equations (5)–(7) and the non-linear forms as given in Equations (8)–(10) [24], by using Origin 8.5 software (OriginLab Corp., Northampton, MA, USA):

(5) $$\frac{C_{e}}{q_{e}}=\frac{1}{\left(q_{m}\times K_{L}\right)}+\frac{1}{q_{m}}{\;C}_{e}$$

(6) $$\log q_{e}=\frac{1}{n}\log C_{e}+\log K_{F}$$

(7) $$q_{e}={B\;\mathrm{lnK}}_{T}+{B\;\mathrm{lnC}}_{e}$$

(8) $$q_{e}=\frac{C_{e}{\;q}_{m}{\;K}_{L}}{1+K_{L}C_{e}}$$

(9) $$q_{e}=K_{F}{\;C}_{e}{}^{1/n}$$

(10) $$q_{e}=q_{m}\ln\left(K_{T}C_{e}\right)$$

where q_e_ is the amount of solute adsorbed at equilibrium per adsorbent unit weight (mg·g^−1^), C_e_ represents the concentration of solute at equilibrium (mg·L^−1^), q_m_ represents the maximum adsorption capacity (mg g^−1^), K_L_ is the Langmuir isotherm constant (L mg^−1^), K_F_ is the Freundlich isotherm constant (L g^−1^), n is the coefficient of the Freundlich equation related to heterogeneity (measure of adsorption linearity), B is related to sorption heat and K_T_ (L mg^−1^) is Temkin isotherm equilibrium binding constant. The sum of squares error (SSE) is employed to estimate the goodness of non-linear fit. The best model is characterized by the smallest SSE value.

The Dubinin–Radushkevich isotherm model was also evaluated by using the Equation (11):

(11) $$\ln\;q_{e}=\ln q_{m}-{\beta \;\varepsilon }^{2},$$

where

(12) $$\varepsilon =\mathrm{RT}\;\ln\left(1+1/C_{e}\right),$$

where q_m_ (mg Zn g^−1^) is the maximum adsorption capacity, β (mol^2^ kJ^−2^) is a coefficient related to the mean sorption energy, ε is the Polanyi potential, R (J mol^−1^ K^−1^) is the gas constant and T (K) is the absolute temperature. The sorption energy E (kJ mol^−1^) is given by:

(13) $$E=1/\sqrt{-2\beta }$$

The fittings of the experimental data collected to three different kinetics models aimed to study the adsorption rate and the rate-controlling step during the removal of the heavy metal ions. The first two models considered, PFO and PSO, are reaction kinetics models that are usually adequate for cases in which there is a high and low concentration of the pollutant in the solution, respectively. In the PSO model, chemisorption is supposed to be the rate-controlling step in the process. In the third model considered, the rate-controlling step is the intraparticle diffusion of the pollutant into the pores of the adsorbent. This model is usually adequate for porous adsorbents [25,26].

## 3. Results

### 3.1. Adsorbents Characterization

#### 3.1.1. Chemical Composition of Lignocellulosic Biomass

In wood, the major component is cellulose and lignin [19]. Studies performed during the last decades [19,20], showed that lignin plays a significant role in the removal of heavy metals due to the presence of phenolic groups. However, this is not the predominant factor, since there are other components such as hemicellulose that have ionizable carboxylic groups in their composition that contribute to the adsorption of metal ions. As shown in Table 1, the values in this study, vary between 63 and 74% for cellulose, while lignin content varied from 18 to 32%.

#### 3.1.2. Infrared Spectroscopy

**Figure 1:**
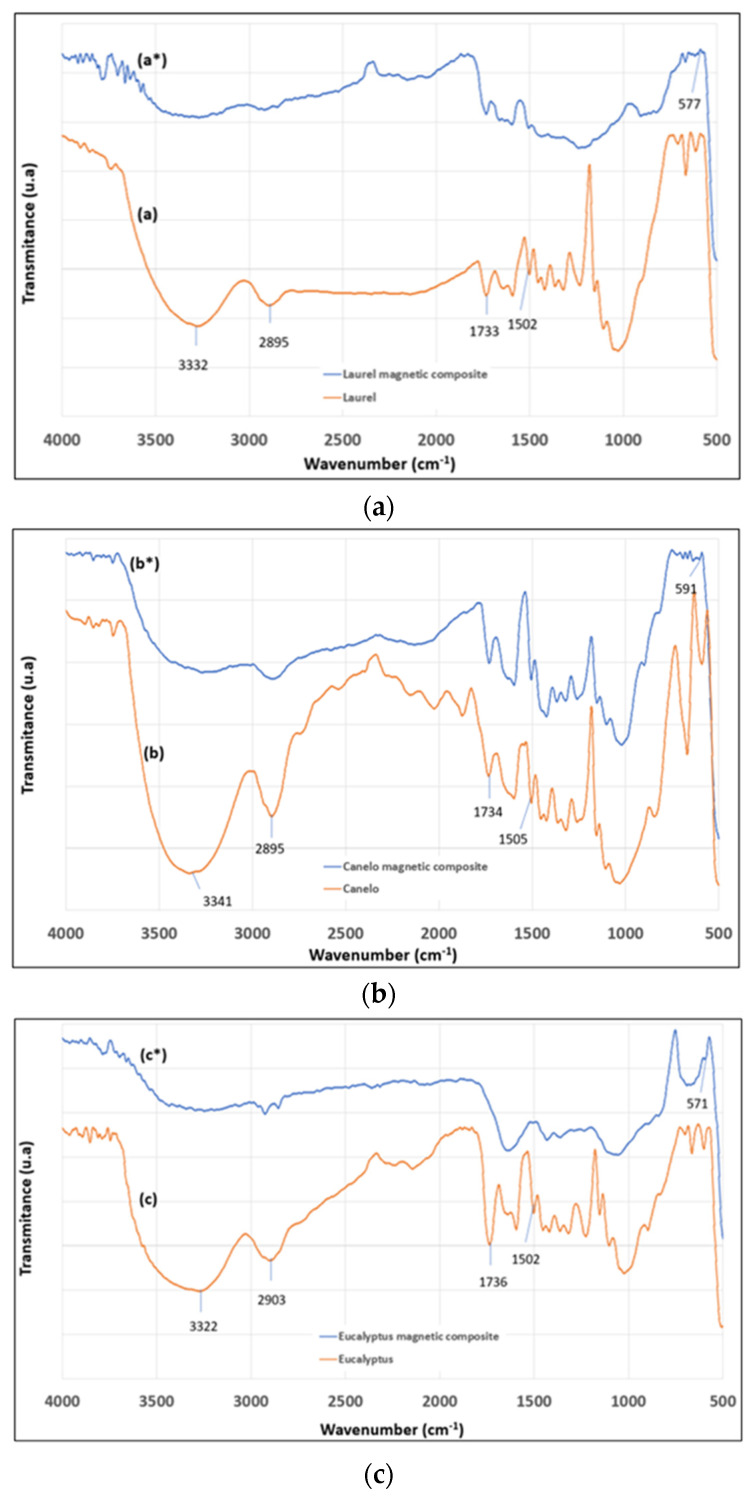
FTIR spectra of lignocellulosic biomass before (x) and after impregnation with magnetite (x*): (**a**) (a* vs. a) laurel, (**b**) (b* vs. b) canelo and (**c**) (c* vs. c) eucalyptus.

Figure 1 shows FTIR spectra of the lignocellulosic residues and the magnetic composites. The laurel, canelo and eucalyptus residues showed similar spectra to literature [27] with bands located at 3332, 3341 and 3322 cm^−1^, respectively, which correspond to O–H bond stretches of phenol and alcohol groups. The bands at 2895, 2895 and 2903 cm^−1^ attributed to the C–H bonds stretches of the aliphatic chains, while 1733, 1734 and 1736 cm^−1^ bands correspond to C=O groups [28]. Characteristic bands of lignin for laurel, canelo and eucalyptus were seen at 1502, 1505 and 1502 cm^−1^, respectively, which correspond to the benzene ring vibration. Bands at 1733, 1734 and 1736 cm^−1^ are attributed to the carbonyl group vibration of cellulose and hemicellulose. Bands between 1154 and 1024 cm^−1^ represent the vibrational stretching of the C–OH groups. Although very weak, the bands at 577, 591 and 571 cm^−1^, respectively in the magnetic composites of laurel, canelo and eucalyptus could be attributed to the Fe–O bond in magnetite [5,12]. Such weak bands could be attributed to the region of the sample studied in ATR mode. Nevertheless, their presence could be also retrieved by a slight shift in the biomass bands upon modification. Moreover, as the microscopy and diffraction measurements further show, the presence of magnetite is confirmed.

#### 3.1.3. Scanning and Transmission Electron Microscopy

**Figure 2:**
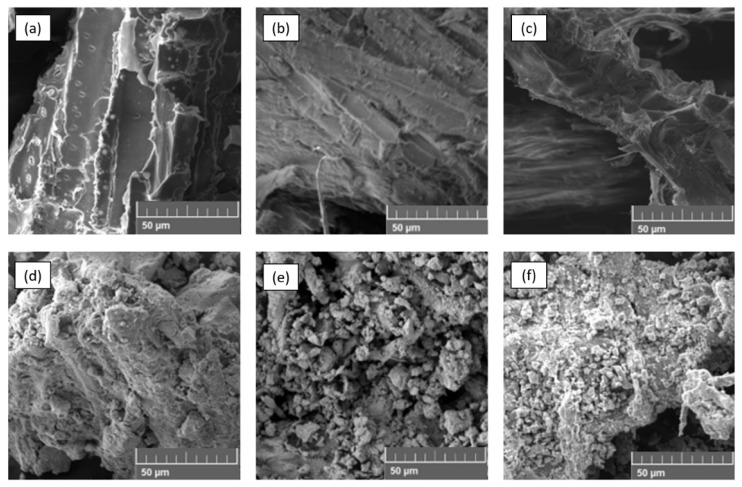
SEM image of non-impregnated lignocellulosic biomass from: (**a**) eucalyptus, (**b**) canelo and (**c**) laurel, and their magnetite impregnated counterparts: (**d**–**f**).

**Figure 3:**
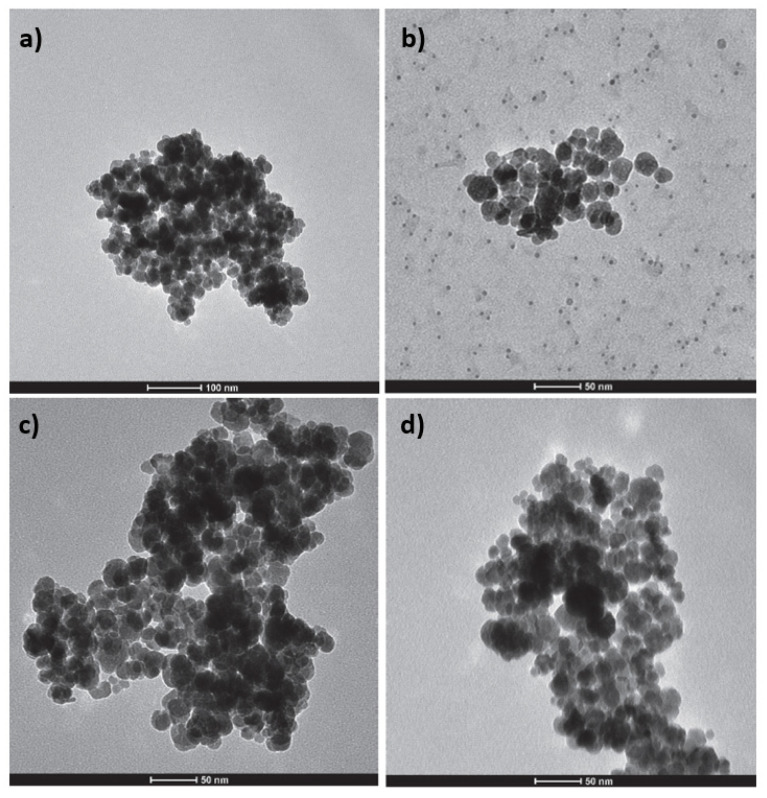
TEM images of the magnetite nanoparticles (**a**) and magnetite modified sorbent: canelo (**b**), eucalyptus (**c**) and laurel (**d**).

Figure 2 shows SEM images of lignocellulosic residues and composite materials. Non-homogeneous surfaces with smooth sections are seen in the lignocellulosic residues. Magnetic composites show rough surfaces due to the presence of magnetite nanoparticles deposited on the lignocellulosic residue surfaces. Agglomerates of different size are formed due to the magnetic properties of the nanoparticles and the high surface area [29]. TEM images of magnetite particles (a) and magnetic composite of laurel (b) are shown in Figure 3. These pictures reveal nanoscaled quasi-spherical particles with particle diameter of 20 nm, approximately. In previous studies [6,30,31], for similar synthesis methods of magnetite nanoparticles, particles with size between 10 and 50 nm and a similar geometry to this study were obtained [32]. Furthermore, according to Shahid and Choi, the dissolution-recrystallization, occurring during coprecipitation process is responsible for the spherical shape of the magnetite nanoparticles [32]. An aggregation phenomenon is evident in the magnetite nanoparticles, which could be attributed to the small size and their magnetic properties. This phenomenon seems to be reduced in the composites as the particles are distributed on the lignocellulosic matrix surface.

#### 3.1.4. X-ray Diffraction

**Figure 4:**
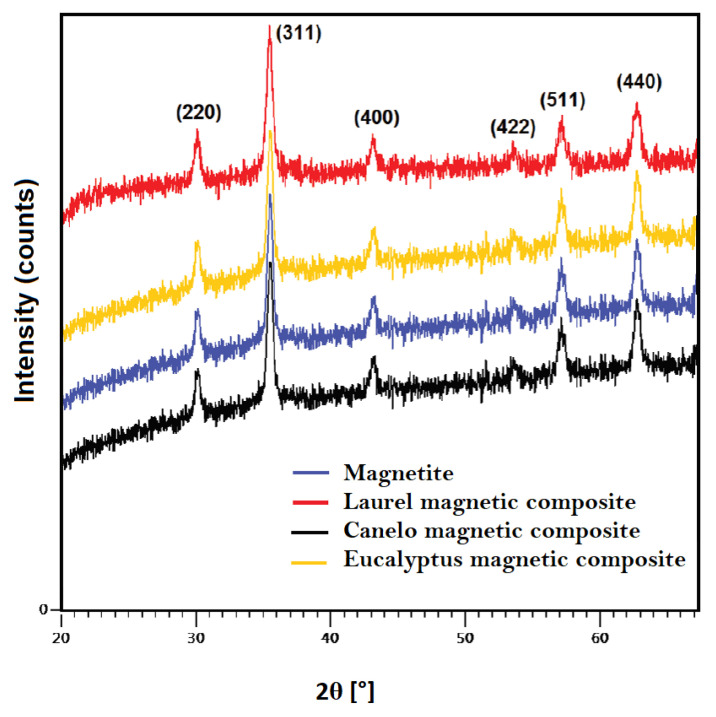
X-ray diffraction pattern of magnetite nanoparticles and magnetic composites.

Figure 4 shows the X-ray diffraction patterns of magnetite and magnetic composites. Peaks at 30.0°, 35.5°, 43.0°, 53.5°, 57.0° and 62.5° corresponding to indices (220), (311), (400), (422), (511) and (440), were observed. These peaks correspond to a cubic spinel structure of magnetite [29]. Based on these results, the presence of magnetite in the composite material can be verified. The same behavior was observed for canelo and eucalyptus magnetic composites. According to the Scherrer equation mentioned elsewhere [33], the crystallite size calculated for magnetite nanoparticles, and magnetite modified laurel, canelo and eucalyptus composites were 10 nm, 15.3 nm, 19.5 nm and 13.5 nm, respectively. These results are comparable to the sizes observed with TEM and reported scale values [32].

### 3.2. Adsorption Studies

#### 3.2.1. Optimal Time and Adsorbent Dosage

**Figure 5:**
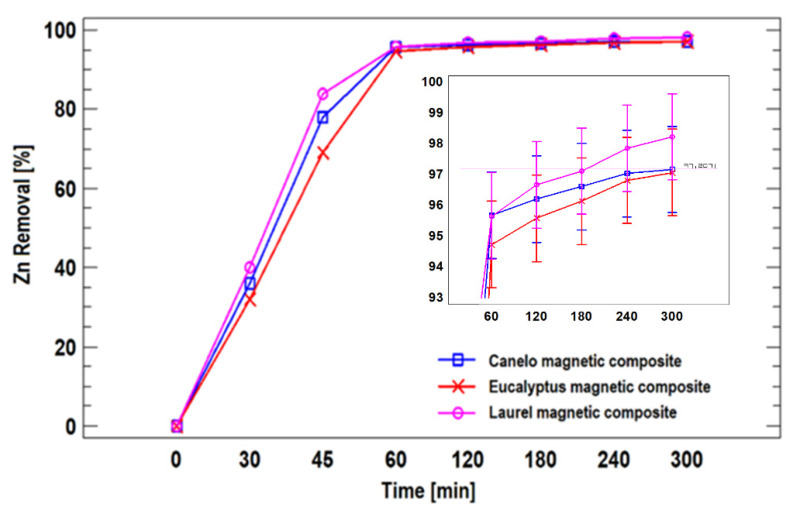
Contact time effect on zinc removal using magnetic composites.

The effect of contact time on adsorption of zinc was studied. As shown in Figure 5, the three magnetic composites have the same behavior. The adsorption rate was fast for the first minutes, after 60 min this rate decreases significantly. According to Zhang et al. [34], during the first few minutes, the adsorbent has many free active sites, and as the adsorption process progresses they are occupied by zinc ions. Furthermore, they generate repulsive forces towards the ions still present in the treated solution, which makes it difficult to occupy all the active sites on the adsorbent surface.

It was observed that after 60 min the Zn(II) removal exceeds 95% for the three composite materials and the equilibrium was achieved after 180 min. This time was considered as the optimal time for the next tests.

**Figure 6:**
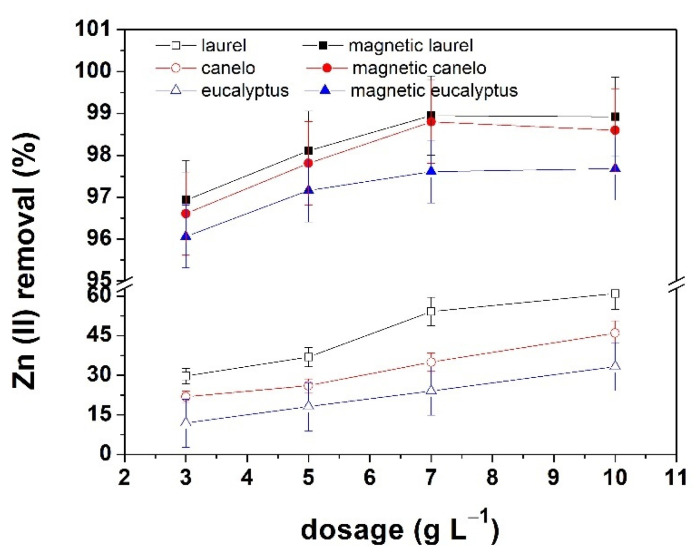
Effect of adsorbent dosage on zinc removal using lignocellulosic biomass before and after magnetite impregnation.

Figure 6 shows the effect of adsorbent dosage on zinc removal from synthetic solutions. It is observed for the three lignocellulosic residues (left) that the dosage has a direct influence in the adsorption efficiency. Laurel sawdust showed the maximum removal percentage (60.9%) of the three residues, which could be attributed to the lignin content of the lignocellulosic residue. For the magnetic composites (right), it is observed a maximum removal of 98.9, 98.8 and 97.6% for laurel, canelo and eucalyptus magnetic composites, respectively, when using 7 g L^−1^ of adsorbent. By using higher dosages of adsorbent (10 g L^−1^), the removal percentage does not increase which is attributed to a higher dispute over available active sites. Thus, the number of agglomerates increases, which causes the diffusion path to be longer and the adsorption process gets hindered [13].

When comparing the results obtained in the zinc removal, it is observed that the addition of magnetite nanoparticles to lignocellulosic residues improve notably the removal percentage. For laurel, the zinc removal increases from 60.9 to 98.9%. This behavior occurs due to the presence of nanoparticles, which considerably increases the surface area. Furthermore, the Fe and O atoms of the nanoparticles adsorb OH^–^ radicals from the solution, causing their surface to become rich in OH^–^ groups. This increases the interactions between these groups and the metal ions in the solution, which makes the removal process more efficient [31].

#### 3.2.2. Adsorption Kinetics

**Figure 7:**
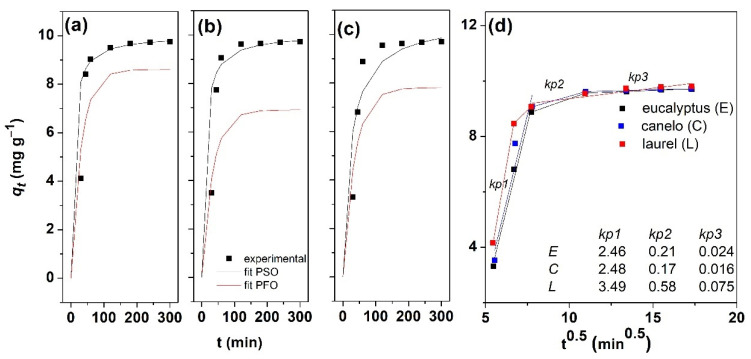
Adsorption kinetics of Zn on magnetite modified lignocellulosic waste-based adsorbents: laurel (**a**), canelo (**b**) and eucalyptus (**c**). Weber–Morris plot for intraparticle diffusion kinetics of Zn adsorption on magnetite eucalyptus modified adsorbent (**d**).

As shown in Figure 7a–c, the Zn adsorption rates on the magnetite modified canelo, laurel and eucalyptus waste-based adsorbents increased rapidly in the initial stage and then gradually decreased reaching an equilibrium state. This behavior could be explained by the existence of numerous accessible active sites on the adsorbent surface and thus, when they get occupied, the adsorption rate decreases. The kinetics of the removal of Zn(II) ions using magnetite modified adsorbents was fitted by both pseudo-first order model (PFO) and pseudo-second order model (PSO) and the corresponding plots are given in Figure 7a–c. As it is clearly observed, the fitted values of *q* were closer to the experimental ones for PSO kinetic model for magnetite modified laurel and canelo adsorbents, which is indicative of the suitability of PSO model for the adsorption kinetics of Zn on the obtained bio-sorbents.

The parameters corresponding to the fitting of kinetic models are further given in the Table 2. The correlation coefficient, R^2^, obtained from the linear regression analysis was used to analyze the linear forms for PFO and PSO models. Thus, the higher values of R^2^ for PSO with respect to the PFO ones (above 0.963), confirm a better fit of the adsorption process by PSO model, independently of the employed adsorbent. This indicates that Zn adsorption process on each magnetic adsorbent could be controlled by chemisorption [35,36], which is also supported by the results of the adsorbent characterization (presence of active sites/functional groups). The fitted adsorption capacities are similar for the modified adsorbents, reaching a value of about 10 mg g^−1^. On the other hand, the parameter *h* increases from eucalyptus to canelo and laurel magnetite-modified adsorbents, indicating the initial adsorption has the highest rate for laurel-based adsorbent. Such results could be attributed to the biomass structure and functional groups [37]. The adsorption rate constants obtained from the plots were decreasing from 0.0141, 0.0116 and 0.0041 g mg^−1^ min^−1^ for laurel, canelo and eucalyptus, respectively.

Despite the good fit of PSO for laurel and canelo based sorbents, the low R^2^ values obtained for eucalyptus sorbent for both PFO and PSO models suggest that a different model could fit the adsorption behavior of the obtained materials, as the sorbent supports have similar characteristics. Thus, intraparticle diffusion model as proposed by Weber and Morris was employed. This model assumes that the rate is not limited by mass transfer from the bulk solution to the external surface of adsorbent as the solution is stirred during the sorption period [38]. Thus, the corresponding plot would be linear [39]. Figure 7d shows multi-linear plots for all sorbents, where the first step is attributed to fast (instantaneous) adsorption of Zn on the sorbent surface, the second step is characterized by a slower sorption due to diffusion of Zn ions into the inner adsorption sites of the adsorbent material, that would be the rate-limiting stage and a third step which corresponds to the equilibrium when the sorbent gets saturated with the ions. This behavior is similar to that of other adsorbents in literature [40,41]. As it can be observed in Figure 7c (kp, mg g^−1^ min^−0.5^), the intra-particle diffusion rate constant for the first step is the highest for laurel and followed by canelo and eucalyptus with similar values, that is initial adsorption process is faster on laurel-based sorbent. The second and third steps are characterized by similar trend of the constants. Since the constant value was markedly higher for the first step than the other two, the boundary diffusion is indicated as dominating the process followed by the intra-particle diffusion.

#### 3.2.3. Adsorption Isotherms

**Figure 8:**
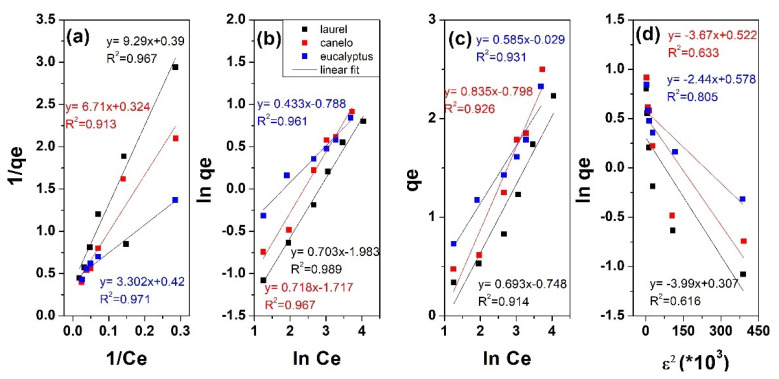
Linear regression analysis of Zn ions adsorption of magnetite modified adsorbents by Langmuir (**a**); Freundlich (**b**); Temkin (**c**) and Dubinin–Radushkevich (**d**), (ε^2^ value multiplied with 10^3^) models.

In order to better understand the adsorption of Zn ions on the obtained magnetite modified adsorbents, linearized forms of Langmuir, Freundlich and Temkin models were used for fitting. The corresponding plots are showed in Figure 8, respectively. Langmuir model is employed for homogeneous adsorption which assumes monolayer adsorption in the absence of interaction or steric hindrance between adsorbed molecules. The D-R model considers on the other hand a porous structure of adsorbent with heterogeneous surface. The Freundlich model is suitable for the adsorption on heterogeneous surfaces. The Temkin model assumes uniform binding energies and that the sorption heat decreases linearly as the sorbent surface gets covered with the ions.

As it can be observed from Figure 8, the Langmuir, Freundlich and Temkin fittings resulted in correlation coefficient R^2^ above 0.9. The adsorption parameters from the linear fitting are further presented in Table 3.

By comparing the correlation coefficient values, one can observe larger values above 0.96 for Freundlich model which indicates better fitting with this model, independently of adsorbent. This suggests the Zn adsorption on the magnetite modified laurel, canelo and eucalyptus adsorbents takes place via multilayer adsorption on heterogeneous surfaces where active sites have different adsorption capacity. The fitting parameters show Freundlich constant (adsorption intensity) decreasing in the order eucalyptus > canelo > laurel for the magnetite modified adsorbents along with increasing *n* value, and thus indicating the Zn adsorption is more favorable on eucalyptus modified adsorbent.

From Langmuir fitting, it can be seen that q_m_ slightly decreased from eucalyptus to laurel along with an increase in K_L_ value. Temkin model can be employed in order to explain the interactions between Zn ions and the sorbent in regard to the heat of adsorption indicated by the B parameter. It was observed that K_T_ was the highest for eucalyptus and laurel, while B decreased in the order eucalyptus, canelo, laurel magnetite-modified adsorbents, indicating physical adsorption process.

The order of the isotherm best fits in this work is Freundlich > Langmuir > Temkin. Although such isotherms are widely employed, they do not give information in regards to the mechanism of sorption. For this purpose, the Dubinin–Radushkevich (D-R) isotherm model employed for adsorption process taking place on both homogeneous and heterogeneous surfaces was further applied for modelling the adsorption process (see Table 3 for fitting parameters). The correlation fitting R^2^ value is below 0.8, indicating poor fitting with this model. The maximum adsorbed amount decreased from eucalyptus to laurel sorbent while and the sorption energy decreased in the same order and indicated a physical nature of the adsorption process [42]. The X_m_ value is slightly smaller than q_m_ which could be attributed to the different definition of q_m_ in the models.

**Figure 9:**
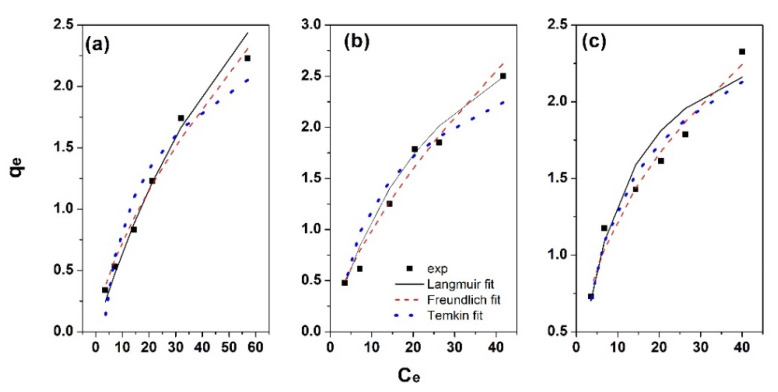
Non-linear regression models of Zn ions adsorption on magnetite modified adsorbents: laurel (**a**), canelo (**b**) and eucalyptus (**c**).

On the other hand, given the errors introduced by the linearized forms of the isotherm models, non-linear regression is preferable and more relevant to estimate the isotherm model parameters. The plots are represented in Figure 9 showing the fitting curves for each model with respect to the experimental data. The fitting parameters are depicted in Table 4. It can be observed that the non-linear regression resulted in R^2^ values similar to those from linear regression. The SSE values decreased in the order Temkin < Langmuir < Freundlich for all sorbents. As the best fit is indicated by lower SSE values, it can be concluded that Freundlich non-linear model best fits the adsorption on the obtained materials.

To verify the suitability of the magnetite modified composite as adsorbents, a comparison with reports on other similar adsorbents in the literature was performed. Although direct comparison of the maximum adsorption capacity q_m_ of studied lignocellulosic biomass-based adsorbents with literature is difficult, owing to the different experimental conditions, the maximum adsorption capacity obtained is in line with other reports on either same biomass sorbents (different analyte) [43] or adsorbents for Zn(II) obtained from various biomass such as Lagenaria vulgaris shell [44], modified orange peel [45] or [46] or magnetic water hyacinth [47]. Considering these findings and the cost-effective source of the obtained adsorbents, the laurel, canelo and eucalyptus biomass is considered a promising alternative to obtain highly effective adsorbents for water treatment.

## 4. Conclusions

Laurel, canelo and eucalyptus residues were used as support of magnetic composites to be used as adsorbents in zinc removal from synthetic solutions. XRD, FTIR, SEM and TEM analysis showed that magnetite nanoparticles with diameters of approximately 20 nm were obtained and that were effectively impregnated in lignocellulosic matrices. Impregnation of magnetite in the lignocellulosic matrices of laurel, canelo and eucalyptus increased the zinc removal in 79.7, 172.3 and 282.2%, respectively, in comparison to the lignocellulosic residues alone. The optimal contact time was 3 h and an adsorbent dosage of 7 g/L allowed the maximum removal of zinc, which was about 98.9, 98.8 and 97.6% for laurel, canelo and eucalyptus magnetic composites, respectively. The adsorption of zinc on magnetite modified adsorbents is best fit by the isotherm models: Freundlich > Langmuir > Temkin > Dubinin–Radushkevich, indicating multilayer adsorption on heterogeneous surfaces while kinetics models shown the adsorption process is dominated by both the chemisorption and diffusion.

## Figures and Tables

**Table 1:** Chemical composition and proximate analysis of the lignocellulosic biomass.

	Component	Laurel	Canelo	Eucalyptus
Chemical composition	Cellulose [wt %]	50.20	53.49	58.44
	Lignin [wt %]	31.84	28.28	18.28
	Hemicellulose [wt %]	12.76	12.45	16.01
	Extractives [wt %]	5.20	5.78	7.27
Proximate analysis	Volatile matter [wt %]	70.94	78.70	81.52
	Fixed carbon [wt %]	17.99	12.93	12.54
	Ash content [wt %]	1.38	0.16	0.25
	Moisture [wt %]	9.69	8.21	5.69

**Table 2:** Kinetics parameters for Zn(II) ions removal using the magnetite impregnated adsorbents.

Lignocellulosic Biomass	Pseudo First-Order			Pseudo Second-Order			
	$q_{e\;}$ $\left[\frac{mg\;Zn}{g}\right]$	$k_{1}\;$ $\left[min^{-1}\right]$	R^2^	$q_{e\;}$ $\left[\frac{mg\;Zn}{g}\right]$	$k_{2}\;$ $\left[\frac{g}{mg\;Zn\times min}\right]$	h $\left[\frac{mg\;Zn}{g\times min}\right]$	R^2^
Laurel	8.602	0.0323	0.873	10.013	0.0141	1.412	0.998
Canelo	6.913	0.0296	0.946	10.052	0.0116	1.171	0.999
Eucalyptus	7.81	0.0275	0.947	10.613	0.0041	0.456	0.963

**Table 3:** Linear adsorption isotherm fitting parameters on magnetic adsorbents.

Isotherm Model/Adsorbent	Parameter	Laurel	Canelo	Eucalyptus
Langmuir	q_m_, mg g^−1^	0.11	0.15	0.31
	K_L_ (L mg^−1^)	23.8	20.7	7.9
	R^2^	0.967	0.913	0.971
Freundlich	q_m_, mg g^−1^	2.31	2.61	2.18
	K_F,_ (L g^−1^)	0.12	0.18	0.5
	n	1.25	1.39	2.31
	R^2^	0.989	0.967	0.961
Temkin	K_T_, (L mg^−1^)	0.89	0.38	0.95
	B	0.69	0.84	0.58
	R^2^	0.914	0.926	0.931
D-R	Xm, (mg g^−1^)	1.36	1.68	1.78
	E, (kJ mol^−1^)	0.353	0.37	0.452
	R^2^	0.616	0.633	0.805

**Table 4:** Non-linear adsorption isotherm fitting parameters on magnetic adsorbents.

Isotherm Model/Adsorbent	Parameter	Laurel	Canelo	Eucalyptus
Langmuir	q_m_, mg g^−1^	6	4.2	2.7
	K_L_ (L mg^−1^)	0.012	0.035	0.1
	R^2^	0.971	0.956	0.978
	SSE	0.065	0.1	0.078
Freundlich	q_m_, mg g^−1^	2.31	2.62	2.24
	K_F,_ (L g^−1^)	0.16	0.21	0.454
	n	1.52	1.48	2.31
	R^2^	0.989	0.972	0.966
	SSE	0.046	0.08	0.039
Temkin	K_T_, (L mg^−1^)	0.34	0.54	0.951
	B	0.693	0.72	0.585
	R^2^	0.914	0.921	0.931
	SSE	0.182	0.248	0.081

## Data Availability

The data presented in this study are available on request from the corresponding author.
